# Geo-spatial factors associated with infection risk among young children in rural Ghana: a secondary spatial analysis

**DOI:** 10.1186/s12936-016-1388-1

**Published:** 2016-07-08

**Authors:** Ashley M. Aimone, Patrick E. Brown, Stanley H. Zlotkin, Donald C. Cole, Seth Owusu-Agyei

**Affiliations:** Division of Epidemiology, Dalla Lana School of Public Health, University of Toronto, 155 College Street, Toronto, ON M5T 3M7 Canada; Division of Biostatistics, Dalla Lana School of Public Health, University of Toronto, 155 College Street, Toronto, ON M5T 3M7 Canada; Centre for Global Child Health, Hospital for Sick Children, Peter Gilgan Centre for Research and Learning, 686 Bay Street, Toronto, ON M5G 0A4 Canada; Kintampo Health Research Centre, Kintampo, Ghana

**Keywords:** Spatial, Infection, Malaria, Children, Geostatistical modelling, Bayesian inference

## Abstract

**Background:**

Determining the spatial patterns of infection among young children living in a malaria-endemic area may provide a means of locating high-risk populations who could benefit from additional resources for treatment and improved access to healthcare. The objective of this secondary analysis of baseline data from a cluster-randomized trial among 1943 young Ghanaian children (6–35 months of age) was to determine the geo-spatial factors associated with malaria and non-malaria infection status.

**Methods:**

Spatial analyses were conducted using a generalized linear geostatistical model with a Matern spatial correlation function and four definitions of infection status using different combinations of inflammation (C-reactive protein, CRP > 5 mg/L) and malaria parasitaemia (with or without fever). Potentially informative variables were included in a final model through a series of modelling steps, including: individual-level variables (Model 1); household-level variables (Model 2); and, satellite-derived spatial variables (Model 3). A final (Model 4) and maximal model (Model 5) included a set of selected covariates from Models 1 to 3.

**Results:**

The final models indicated that children with inflammation (CRP > 5 mg/L) and/or any evidence of malaria parasitaemia at baseline were more likely to be under 2 years of age, stunted, wasted, live further from a health facility, live at a lower elevation, have less educated mothers, and higher ferritin concentrations (corrected for inflammation) compared to children without inflammation or parasitaemia. Similar results were found when infection was defined as clinical malaria or parasitaemia with/without fever (definitions 3 and 4). Conversely, when infection was defined using CRP only, all covariates were non-significant with the exception of baseline ferritin concentration. In Model 5, all infection definitions that included parasitaemia demonstrated a significant interaction between normalized difference vegetation index and land cover type. Maps of the predicted infection probabilities and spatial random effect showed defined high- and low-risk areas that tended to coincide with elevation and cluster around villages.

**Conclusions:**

The risk of infection among young children in a malaria-endemic area may have a predictable spatial pattern which is associated with geographical characteristics, such as elevation and distance to a health facility.

*Original trial registration* clinicaltrials.gov (NCT01001871)

**Electronic supplementary material:**

The online version of this article (doi:10.1186/s12936-016-1388-1) contains supplementary material, which is available to authorized users.

## Background

According to the World Health Organization (WHO), the leading causes of death in children under 5 years of age are infection-related—primarily pneumonia, diarrhoea and malaria—and approximately 45 % of all deaths are associated with malnutrition [1]. Child mortality rates are highest in low- and middle-income countries (LMICs), particularly in sub-Saharan Africa where the risk of death is 15 times greater than in high-income regions [1]. Malnourished children are more vulnerable to infections, primarily due to compromised immune function and epithelial integrity and inflammation [2]. For example, Muller et al. [3] reported a positive association between malaria morbidity and the degree of protein-energy malnutrition among children in West Africa. Micronutrient deficiencies also have a compromising effect on immune function, which can usually be improved through diet changes, food fortification or supplementation [4]. However, for iron nutrition, the relationship between iron deficiency and infection risk is less clear. Evidence suggests that providing iron as a supplement or through fortification to children with high infection exposure may or may not increase the risk of infection-related morbidity and mortality [5–8]. Conversely, inflammation due to infection can affect iron homeostasis [9] and the risk of iron deficiency [10], particularly in cases of prolonged or chronic infection. Assessing the risk of infection is an important first step in developing safe and effective means of administering iron to children in LMICs where iron deficiency and anaemia are prevalent.

Infection status can be assessed using biomarkers such as C-reactive protein (CRP), an acute phase protein that becomes elevated in response to the early phase of the inflammatory response (approximately 24–48 h) [11]. The feasibility of measuring such indicators among children in a low-resource context is limited, especially at a population level, as they may require relatively large blood samples and sophisticated analytical methods with laboratory equipment. As such, there are clear advantages in identifying indicators or risk factors associated with infection in LMICs that are not invasive or costly to measure, and thus provide a more feasible means of identifying high-risk populations. This need could be addressed with geographical factors (or ‘geo-indicators’), as the environmental or spatial characteristics of a village or region could provide insight into the dynamics and distribution of infection risk among children. Collecting geo-spatial data is non-invasive and less costly compared to biological measures and they are often publicly available, which improves the access to and comparability of population-level statistics across regional and national borders.

There is mounting evidence to support the use of geographical information systems (GIS) and spatial analysis methods for conducting disease surveillance and risk analysis, assessing health system access and informing health system planning [12–15]. In terms of infectious disease research, there are several examples where geostatistical methods have been used to investigate the spatial patterns and associated risk factors of malaria or other infections among children in LMICs [16–18]. What is lacking, however, is an investigation of the spatial factors associated with childhood morbidity, defined using a combination of malaria infection and inflammatory biomarkers. Even fewer studies have used spatial analysis to link the geographical variation of infection with iron deficiency risk among children in low-resource settings [19]. Soares Magalhaes and colleagues used survey data to build Bayesian geostatistical models to determine the relative contribution of parasitic infections (malaria and helminth) to the spatial variation of anaemia risk among children (≤15 years of age) in northern Angola [19]. The authors found that anaemia, *Plasmodium falciparum* and *Schistosoma haematobium* tended to cluster around inland bodies of water, and estimated that approximately 15.6 and 9.7 % of the spatial variation of anaemia risk was attributable to malaria and schistosomiasis, respectively [19]. While Soares Magalhaes and colleagues provided a good starting point for the integration of infection control programmes with iron supplementation, a drawback of their analyses was the use of anaemia as an indicator of iron status rather than a more specific biomarker, such as ferritin concentration. There are many causes of anaemia in addition to iron deficiency [20].

Considering the bi-directional relationship between infection and iron homeostasis, the ability to describe the spatial variation of infection risk while accounting for iron status may allow us to more confidently identify areas where integrated infection and iron deficiency control programmes are most needed. The objective of the current analysis was to determine the geo-spatial factors associated with malaria and non-malaria infection risk among children with varying levels of iron sufficiency in rural Ghana. The sections that follow include a summary and interpretation of the results of this analysis, as well as a discussion of their contribution to, and implications for, global health research.

## Methods

### Study population

**Fig. 1:**
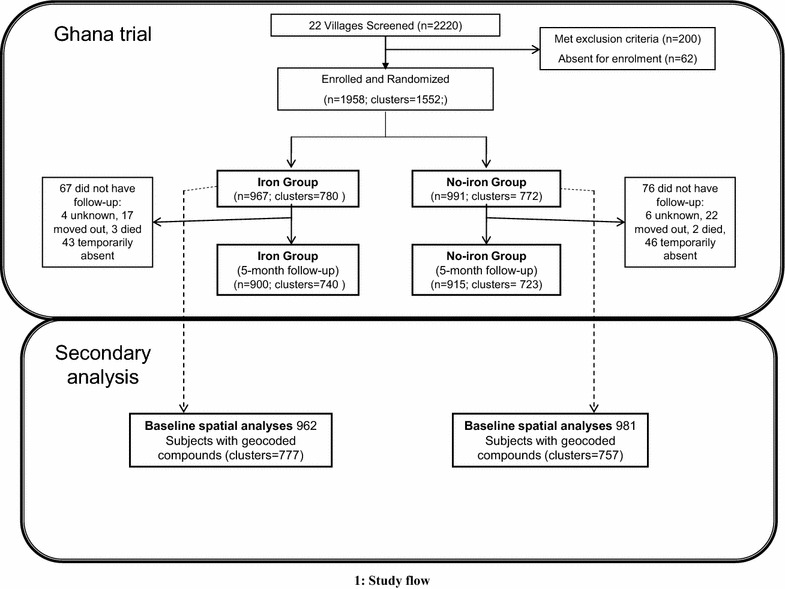
Study flow. Flow of participants through the Ghana trial (*top section*) and secondary analyses (*bottom section*). Out of the 1958 participants from the Ghana trial, a total of 1943 with geocoded compounds were included in the baseline secondary spatial analyses (13 compounds were untraceable, corresponding to 15 participants not included in the secondary analyses)

**Fig. 2:**
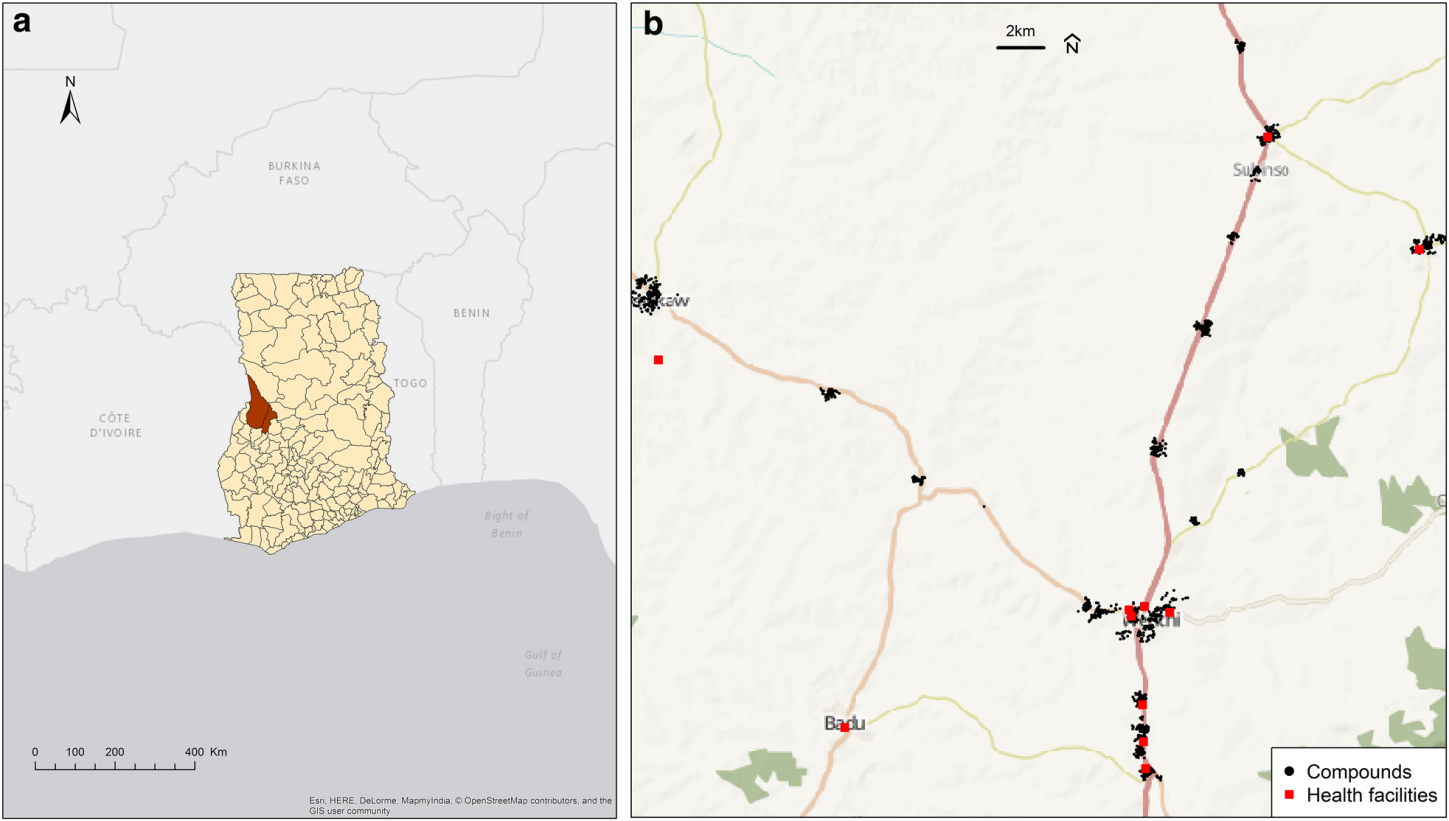
Geographical layout of the trial area. **a** Wenchi and Tain Districts (*red*) in the Brong-Ahafo Region of Ghana. **b** Location of Ghana study compounds (*black points*) and health facilities (*red squares*)

The data used in these analyses were generated from the baseline survey of a community-based, cluster-randomized trial conducted in 2010 in Wenchi and Tain districts of the Brong-Ahafo region, a substantially rural area of Ghana [6]. At the time there were an estimated 7.2 million cases of malaria per year in Ghana, and the prevalence of anaemia among preschool-aged children was 76.1 % (95 % CI 73.9–78.2 %) [21, 22]. Briefly, the aim of the randomized trial was to determine the effect of providing iron with other micronutrients in powder form for 5 months during the rainy season (March–November) on the incidence of malaria among 1958 children aged 6–35 months (representing 1552 clusters and 22 villages) (Fig. 1) [6]. A village was eligible for inclusion in the study if the inhabiting households had at least one child between 6 and 35 months of age. Potentially eligible participants were screened, beginning with villages near the north-east border of Wenchi, then moving to adjacent villages along the main road network. Eligible children were aged 6–35 months, eating solid foods, and living in the study area for at least the following 6 months. Exclusion criteria included severe anaemia (haemoglobin <7.0 g/dL), severe malnutrition (weight-for-length z-score < −3.0), receipt of iron supplements within the past 6 months, or chronic illness (e.g. congenital abnormalities). The geographical layout of the trial area (covering approximately 3800 km^2^), including study compounds, health facilities and road networks, is depicted in Fig. 2.

### Secondary measures from trial data

At baseline, biological samples were collected and analysed for biochemical indicators of iron and infection status, including serum ferritin, C-reactive protein (CRP), and malaria parasite density. Plasma CRP was measured using an immunoturbidimetric method (QuickRead CRP, Orion Diagnostica, Espoo, Finland), and serum ferritin, an enzyme immunoassay (Spectro Ferritin S-22, Ramco Laboratories Inc, Stafford, USA). Thin and thick smears were prepared for malaria parasite speciation and count via microscopy (see Zlotkin et al. [6] for a complete description of biochemical and infection measures). Demographic and nutrition-related information was collected at the household and individual levels, including household assets, maternal education, feeding practices, and child body weight and length. Z-scores for weight-for-length, length-for-age were calculated using the WHO Child Growth Standards [23].

### Geographical coordinates

Handheld global positioning system (GPS) units were used to collect geographical coordinates for over 95 % of the study compounds (representing 1943 trial participants), as well as 22 study villages, and surrounding health facilities and road networks. The GPS coordinates were measured using the WGS 1984 coordinate system and transformed using a universal transverse Mercator (UTM) Zone 30 N projection (EPSG code: 32,630).

### Satellite-derived data

Elevation data were downloaded from the US Geological Survey (USGS) [24], with a spatial resolution of 3 arc-seconds (approximately 90 m). Normalized difference vegetation index (NDVI) data, obtained from the Land Processes Distributed Active Archive Center (LPDAAC) [25], were produced by a spectroradiometer that uses blue, red and near-infrared reflectance to determine vegetation indices for 16-day intervals with a 250-m spatial resolution. Land cover (LC) data (downloaded from worldgrids.org) had a spatial resolution of 500 m, and consisted of 17 land cover classes sub-grouped into three categories: natural vegetation (11 classes), developed and mosaic land (three classes), and non-vegetation (three classes) [26].

### Spatial modelling

The data were analysed using generalized linear geostatistical models (GLGM) [27, 28]. Four definitions of baseline infection status served as the dependent variables: (1) inflammation (CRP > 5 mg/L) and/or malaria parasitaemia; (2) inflammation (CRP > 5 mg/L) without parasitaemia; (3) parasitaemia with measured concurrent fever (axillary temperature >37.5 °C) or reported history of fever within 48 h (i.e., clinical malaria); and, (4) parasitaemia with or without concurrent fever or history of fever. All dependent variables were binary-valued (coded as ‘1’ for positive infection status), and analysed using a logistic model. The four different outcomes were modelled separately in order to explore whether observed geo-spatial associations were influenced by the way infection was defined, and how much of this influence may have been driven by malaria versus non malaria infection types. Geo-spatial and non-spatial variables were chosen for inclusion in the final models based on expert opinion and a review of the literature pertaining to spatial risk factors of malaria and anaemia among young children in low- and middle-income countries [18]. Variables were eligible for inclusion if they were considered to be direct or indirect antecedent factors associated with infection (e.g., elevation), and excluded if they were potential outcomes of infection (e.g., anaemia).

The models were fit using Bayesian inference via an integrated nested laplace approximation (INLA) algorithm [29]. Given the exploratory nature of the analyses, weak or uninformative priors were used for all model parameters with the exception of the Matern shape parameter, which was fixed at 2. Spatial predictions were made on a 100-cell grid covering the study area. The Matern correlation, approximated by a Markov random field [30], extended an additional 3000 m in each direction. Infection probabilities, after transformation with a logit link function, were modelled as the sum of the contributions of the explanatory variables, as well as spatially correlated and compound-level random effect terms. The posterior medians of the odds of infection were computed, assuming baseline values for individual-level covariates and location-specific values for the spatial covariates. A spatially continuous (or geostatistical) model was used for the spatial random effect term, where the correlation between the log-odds of infection of two individuals was given by a Matern spatial correlation function and applied to the distance separating their respective compounds. All spatial modelling was conducted using the *glgm* function from the ‘geostatsp’ package in R [31, 32].

In order to gain additional insight into the variable relationships of interest, five different combinations of selected candidate variables were modelled separately for each outcome. Models 1–3 included independent variables grouped by measurement level. Model 1 included individual-level variables only: baseline child age, sex, weight-for-length z-score and length-for-age z-score, and baseline iron status (ferritin concentration). Age in months was calculated using the reported date of birth and trial enrolment date. The age variable was included in all models with a change point at 24 months, as this was the closest half-year to the mean age of those children who were no longer receiving breast milk (mean = 26.8 months ± 5.8, n = 746). Similar age variable definitions have been used in other studies of iron deficiency and anemia in children [33, 34].

Model 2 included only household-level variables: asset score, maternal education, and distance from each compound to the nearest health facility. Household asset score was generated using a principal component analysis of six economic indicators (farm ownership, size and type of crops grown, type of toilet facility, house ownership). For descriptive purposes, asset score was dichotomized at the median; however, it was modelled continuous variable. Maternal education was included as a binary variable, representing ‘none’ (0) versus ‘any’ (1) level of education (e.g., primary, middle, secondary or higher). Distance to the nearest health facility (an indicator of access to the health care system) was measured ‘as the bird flies’ (straight-line or Euclidean distance) using the near table tool in ArcMap (ArcGIS 10.2, Environmental Systems Resource Institute, Redlands, CA, USA).

Five satellite-derived variables were included in Model 3: elevation, land cover type (LC), NDVI, and two NDVI-LC interaction terms. Elevation was included as a proxy for temperature [35], and ranged across the trial area from 116 to 530 m. elevation values were centred by subtracting 250 m before including them in the analyses. Land cover type was a discrete categorical variable consisting of three values: woody savannah (LC = 8, n = 21/1943 observations), urban and built-up land (LC = 13, n = 243/1943 observations), and cropland/natural vegetation mosaic (LC = 14, n = 1679/1943 observations). In all analyses, the largest category (cropland/natural vegetation mosaic) was used as the reference. Given that the Ghana trial was conducted during the rainy season, rainfall was not expected to vary substantially across the study area, and thus was not included as a spatial variable. Rather, NDVI (a measure of ‘greenness’) was included as an indicator of water accumulation potential or soil moisture [16]. NDVI values were averaged over the year that the study was conducted (2010) in a single raster file, and ranged from 0.22 to 0.62. An interaction term for NDVI and LC was created by, first, using the NDVI raster to mask the LC raster except in areas where LC had a cell value of 8 (woody savannah). The unmasked cells were then given a value of 0. The same method was also used to create the NDVI-LC interaction term for LC values of 13 (urban and built-up land). The new rasters for the interaction terms were then included in the analyses to investigate whether the association between the dependent variable (infection status) and vegetation (or soil moisture) varied across areas with or without a woody savannah or urban/built-up land cover type.

The final model (Model 4) combined selected variables from Models 1–3, including age, sex, weight-for-length z-score, length-for-age z-score, baseline iron status (serum ferritin corrected for CRP using the regression method and re-scaled by multiplying each corrected value by the inverse of the inter-quartile range), asset score, distance to the nearest health facility, and elevation. Variable selection was informed by exploratory descriptive analyses using generalized additive models, linear regression modelling, and simulation analyses. As a confirmatory modelling step, a ‘maximal’ model (Model 5) was also developed and included the same variables as the final model with the addition of maternal education, NDVI, LC, and the two NDVI-LC interaction terms. The maximal model provided an opportunity to investigate variable relationships of interest that were not included in the final model in order to preserve statistical power. As such, there was a higher risk of over-parameterization, and thus the findings from Model 5 were interpreted with caution and used mainly for hypothesis-generation.

In all models with individual-level variables, ferritin concentration was corrected for CRP using a regression-based method (Namaste et al., pers. comm.). The advantage of the regression method is that it can correct ferritin for CRP without requiring the use of pre-determined cut-offs (which can vary across the literature partly due to the detection limits of analytical equipment used) and, therefore, better accounts for the linear relationship between inflammation and ferritin. The first step in the correction approach was to natural logarithm (ln)-transform ferritin, and CRP concentrations to approximate a normal distribution. Zero values for CRP were replaced with a constant, near-zero value (0.02 mg/L) before ln transformation. A linear regression coefficient for CRP was obtained using univariate modelling with ferritin as the outcome. A reference value of 0.104 mg/L, representing little or no inflammation, was subtracted from the ln-CRP concentrations in the regression equation. The reference value was obtained from a meta-analysis of data from the Biomarkers Reflecting Inflammation and Nutrition Determinants of Anemia (BRINDA) study, involving 27,865 pre-school aged children across 15 countries [36]. The correction was then applied only to ln-CRP values that were greater than the ln-CRP reference in order to avoid over-adjustments. The adjusted ferritin equation was calculated by subtracting the influence of CRP as follows:

$${\text{Adjusted ferritin}} = NB \cdot { \hbox{max} }\left( {\frac{{CRP_{obs} }}{{CRP_{ref} }},1} \right)^{{ - \beta_{1} }}$$

where ‘NB’ is the actual value of ferritin, β_1_ is the CRP coefficient, ‘obs’ is the raw observations for CRP, and ‘ref’ is the reference value.

Maps of predicted infection probabilities (odds ratios) and residual spatial variation from the final model (Model 4) were plotted and overlaid with a base map of the trial area. The residual spatial variation plot represented the posterior mean of the spatial random effect, corresponding to the difference between the predicted and expected odds of infection at each location (given the spatial covariate at each location). Individual-level non-spatial variables and effect sizes did not contribute to the plots. For example, an odds ratio of 1.5 indicated that all individuals living at a particular location had a 50 % higher risk of infection compared to similar individuals (e.g. in terms of age, sex, iron status) living in an area where the relative risk was 1.0. On the other hand, if two dissimilar individuals (e.g. with different ages) lived at the same location, they had different infection risks; however, both ratios (e.g. risk divided by ‘typical risk’ for their respective ages) were identical. All model output plots had a spatial resolution of 380 m by 380 m per cell. These plots were visually compared to each other and to relevant satellite-derived maps (e.g., elevation) in order to generate potential explanations for the spatial patterns observed.

## Results

Table 1 shows the baseline characteristics (biochemical measures, anthropometrics, demographics) of 1943 children with geocoded compounds, who were included in this secondary analysis (Fig. 1). The mean age at enrolment was 19.2 months, with 69 % (1348/1943) of participants aged below 24 months. After correcting ferritin concentration for inflammation (CRP) using the regression method, the prevalence of iron deficiency (ferritin <12 µg/L) was 21.4 % (415/1943) at baseline. According to CRP measures and parasite counts, approximately one-third of all children had an infection at baseline (719/1943, 37.0 %). The prevalence of wasting (< −2SD for weight-for-length z-score), and stunting (< −2SD for length-for-age z-score) was 8.1 % (158/1942) and 13.8 % (267/1934), respectively.

**Table 1:** Baseline characteristics of the Ghana trial participants

Trial participants with a geo-coded residence (n)	1943
Males (%)	992 (51.1)
Age at enrolment (months), mean (SD)	19.2 (8.5)
Serum ferritin (µg/L), geometric mean (SD)	35.1 (3.65)
Infection status	
C-reactive protein (mg/L), mean (SD)	3.34 (4.96)
Parasite density (count/µL), geometric mean (SD)	3003.0 (5.35)
Inflammation and/or parasitaemia^a^, n (%)	719 (37.0)
Inflammation without parasitaemia^b^, n (%)	272 (14.0)
Parasitaemia with fever^c^, n (%)	150 (7.72)
All parasitaemia^d^, n (%)	447 (23.0)
Anthropometric status^e^	
Weight-for-length z-score, mean (SD)	−0.63 (0.97)
Length-for-age z-score, mean (SD)	−0.81 (1.21)
Maternal education^f^, n (%)	
None	586 (33.5)
Any	1166 (66.5)
Household asset score^g^, n (%)	
Low	866 (47.5)
High	957 (52.5)
Distance to the nearest health facility (km), mean (SD)	2.57 (2.99)

^a^Inflammation and/or parasitaemia = CRP > 5 mg/L and/or any malaria parasitaemia

^b^Inflammation without parasitaemia = CRP > 5 mg/L without malaria parasitaemia

^c^Parasitaemia with fever = any malaria parasitaemia with concurrent fever (axillary temperature > 37.5 °C) or history of reported fever (within 48 h)

^d^All parasitaemia = any malaria parasitaemia with/without fever

^e^Measured at baseline; z-scores estimated using the WHO Child Growth Standards [23]

^f^ Measured at baseline only; total n = 1752 (74 respondents were not mothers, 117 missing due to incomplete surveys)

^g^ Measured at baseline only; reduced sample size (approximately 1825) due to incomplete surveys and ‘unknown’ responses

The results from Models 1–3 have been included in an additional file (see Additional file 1). Briefly, the definition of infection that included both CRP and parasitaemia seemed to be the most sensitive to covariate associations. In Model 1, age (6–23 months), and baseline iron status were positively associated with infection (CRP > 5 mg/L and/or parasitaemia), while length-for-age z-score, and weight-for-length z-score were negatively associated with infection status (Additional file 1: Table S1). In Model 2, lower maternal education and greater distance to the nearest health facility were associated with positive infection status (Additional file 1: Table S2). The only satellite-derived spatial variable associated with infection in Model 3 was elevation, indicating that lower elevation corresponded with higher infection risk at baseline (Additional file 1: Table S3).

Results from the final models (Model 4) indicated that children with inflammation (CRP > 5 mg/L) and/or any evidence of malaria parasitaemia at baseline were more likely to be between 6 and 23 months of age (OR 1.03, 95 % credible interval (CrI) 1.01, 1.05), approximately 10 % more likely to be stunted or wasted (OR 0.92 for length-for-age z-score and 0.89 for weight-for-length z-score), live farther from a health facility (11 % increased odds of infection for each km) and at a lower elevation (7 % increased odd of infection for every 10 m), and/or have higher ferritin concentration (OR 1.15, 95 % CrI 1.07, 1.24) compared to children without inflammation or parasitaemia (Table 2). Similar results were found when infection was defined as clinical malaria or parasitaemia with/without fever (definitions 3 and 4); however, the magnitude of the association with distance to a health facility increased up to a 20 % greater likelihood of infection with each km of separation. Conversely, when infection was defined using CRP only (without parasitaemia), all covariates were non-significant.

**Table 2:** Results from the *final* spatial models (Model 4) of baseline infection status among 1943 Ghanaian children (2010)

Covariates	Odds ratios (95 % CrI)	Range parameter in km (95 % CrI)	Standard deviations of random effects (95 % CrI)	
			Spatial	Compound
(1) Inflammation and/or parasitaemia				
Intercept	0.504 (0.298, 0.874)	4.559 (1.943, 8.945)	0.506 (0.310, 0.856)	0.007 (0.004, 0.028)
Age per month (months)				
6–23	1.032 (1.011, 1.054)			
24–35	0.963 (0.927, 0.999)			
Sex (male reference)	1.104 (0.902, 1.351)			
Length-for-age z-score	0.915 (0.836, 1.001)			
Weight-for-length z-score	0.886 (0.795, 0.987)			
Asset score	1.041 (0.931, 1.164)			
Distance to health facility (km)	1.107 (1.008, 1.218)			
Elevation (m)	0.993 (0.987, 0.998)			
Baseline iron status	1.150 (1.070, 1.241)			
(2) Inflammation without parasitaemia				
Intercept	0.168 (0.095, 0.301)	7.120 (2.691, 15.54)	0.447 (0.231, 0.871)	0.007 (0.004, 0.028)
Age per month (months)				
6–23	0.995 (0.969, 1.022)			
24–35	0.967 (0.916, 1.018)			
Sex (male reference)	1.163 (0.889, 1.521)			
Length-for-age z-score	1.007(0.893, 1.135)			
Weight-for-length z-score	0.909 (0.789, 1.046)			
Asset score	0.989 (0.859, 1.139)			
Distance to health facility (km)	0.916 (0.825, 1.006)			
Elevation (m)	0.999 (0.993, 1.004)			
Baseline iron status	1.047 (0.947, 1.145)			
(3) Parasitaemia with fever				
Intercept	0.037 (0.014, 0.088)	7.548 (3.080, 14.61)	0.823 (0.452, 1.522)	0.007 (0.028, 0.004)
Age per month (months)				
6–23	1.040 (1.002, 1.081)			
24–35	0.924 (0.856, 0.992)			
Sex (male reference)	1.179 (0.812, 1.716)			
Length-for-age z-score	0.944 (0.796, 1.116)			
Weight-for-length z-score	0.812 (0.659, 0.997)			
Asset score	1.058 (0.849, 1.316)			
Distance to health facility (km)	1.162 (0.993, 1.365)			
Elevation (m)	0.991 (0.981, 0.999)			
Baseline iron status	1.213 (1.098, 1.330)			
(4) All parasitaemia				
Intercept	0.217 (0.110, 0.425)	3.374 (1.552, 6.818)	0.684 (0.433, 1.071)	0.007 (0.004, 0.028)
Age per month (months)				
6–23	1.054 (1.027, 1.081)			
24–35	0.973 (0.932, 1.016)			
Sex (male reference)	1.015 (0.797, 1.293)			
Length-for-age z-score	0.876 (0.785, 0.977)			
Weight-for-length z-score	0.898 (0.787, 1.024)			
Asset score	1.069 (0.927, 1.232)			
Distance to health facility (km)	1.200 (1.066, 1.360)			
Elevation (m)	0.992 (0.985, 1.000)			
Baseline iron status	1.149 (1.064, 1.241)			

Infection status definitions

1) Inflammation and/or parasitaemia (binary): 1 = CRP > 5 mg/L and/or any malaria parasitaemia, 0 = CRP ≤ 5 mg/L and absence of parasitaemia

2) Inflammation without parasitaemia (binary): 1 = CRP > 5 mg/L without malaria parasitaemia, 0 = CRP ≤ 5 mg/L without parasitaemia

3) Parasitaemia with fever (binary): 1 = any malaria parasitaemia with concurrent fever (axillary temperature > 37.5 °C) or history of reported fever (within 48 h), 0 = any malaria parasitaemia without concurrent fever or history of reported fever

4) All parasitaemia (binary): 1 = any malaria parasitaemia with/without fever, 0 = absence of parasitaemia with/without fever

Model prior shape = 1.117, model prior rate = 0.157

Baseline iron status = iron status at baseline, defined as serum ferritin concentration (µg/dL) corrected for CRP using the regression method and re-scaled by multiplying each corrected value by the inverse of the inter-quartile range

*CrI* credible interval

**Fig. 3:**
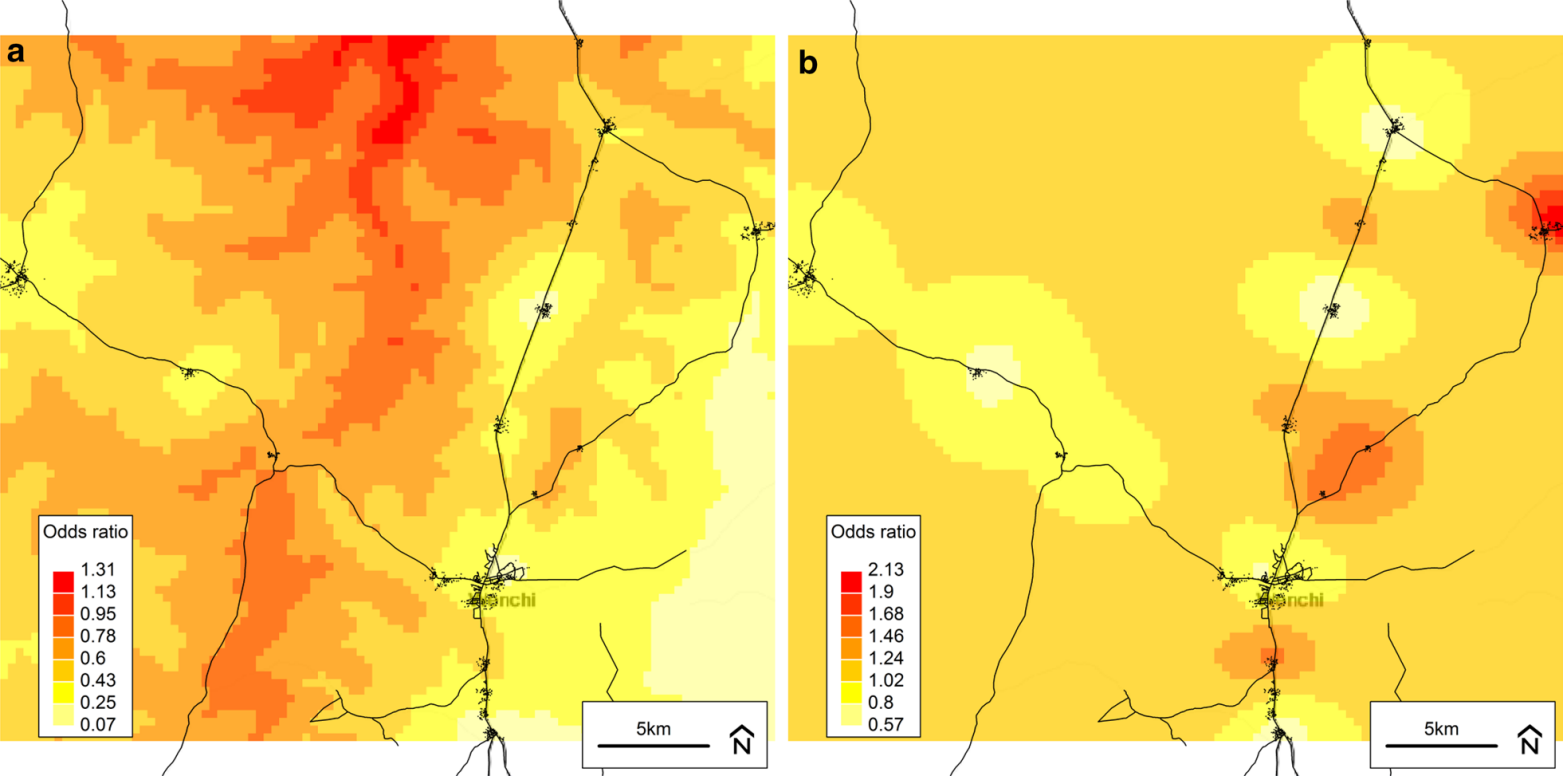
Plots from the final spatial model (Model 4) of inflammation and/or any parasitaemia. **a** Predicted odds of inflammation (CRP > 5 mg/L) *and/or any* malaria parasitaemia at baseline from the final model (Model 4). **b** Residual spatial variation of inflammation (CRP > 5 mg/L) *and/or any* malaria parasitaemia at baseline from the final model (Model 4). *Darker colour* indicates higher risk. Background © Stamen Design

**Fig. 4:**
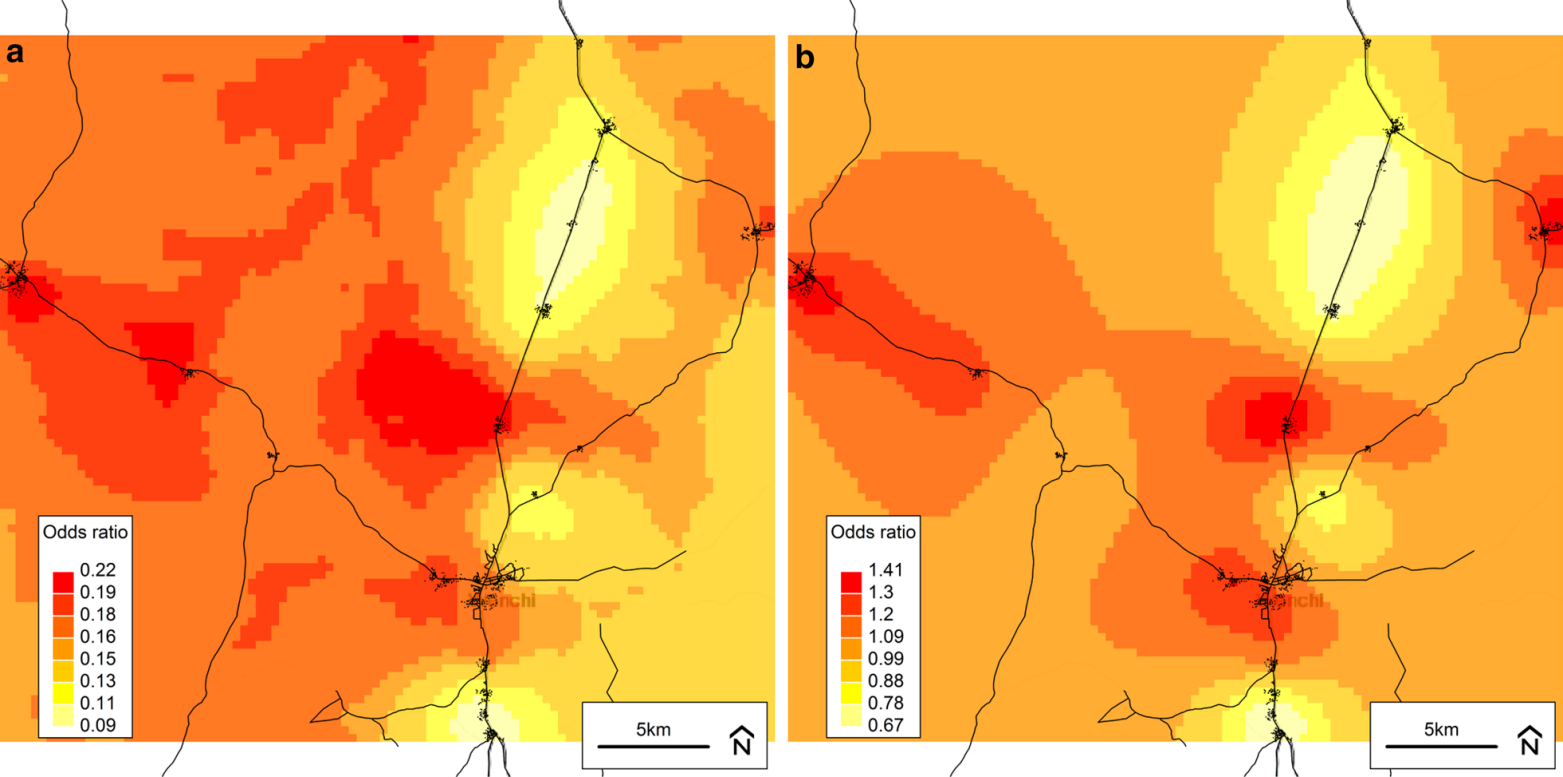
Plots from the final spatial model (Model 4) of inflammation without parasitaemia. **a** Predicted odds of inflammation (CRP > 5 mg/L) *without* malaria parasitaemia at baseline from the final model (Model 4). **b** Residual spatial variation of inflammation (CRP > 5 mg/L) *without* malaria parasitaemia at baseline from the final model (Model 4). *Darker colour* indicates higher risk. Background © Stamen Design

**Fig. 5:**
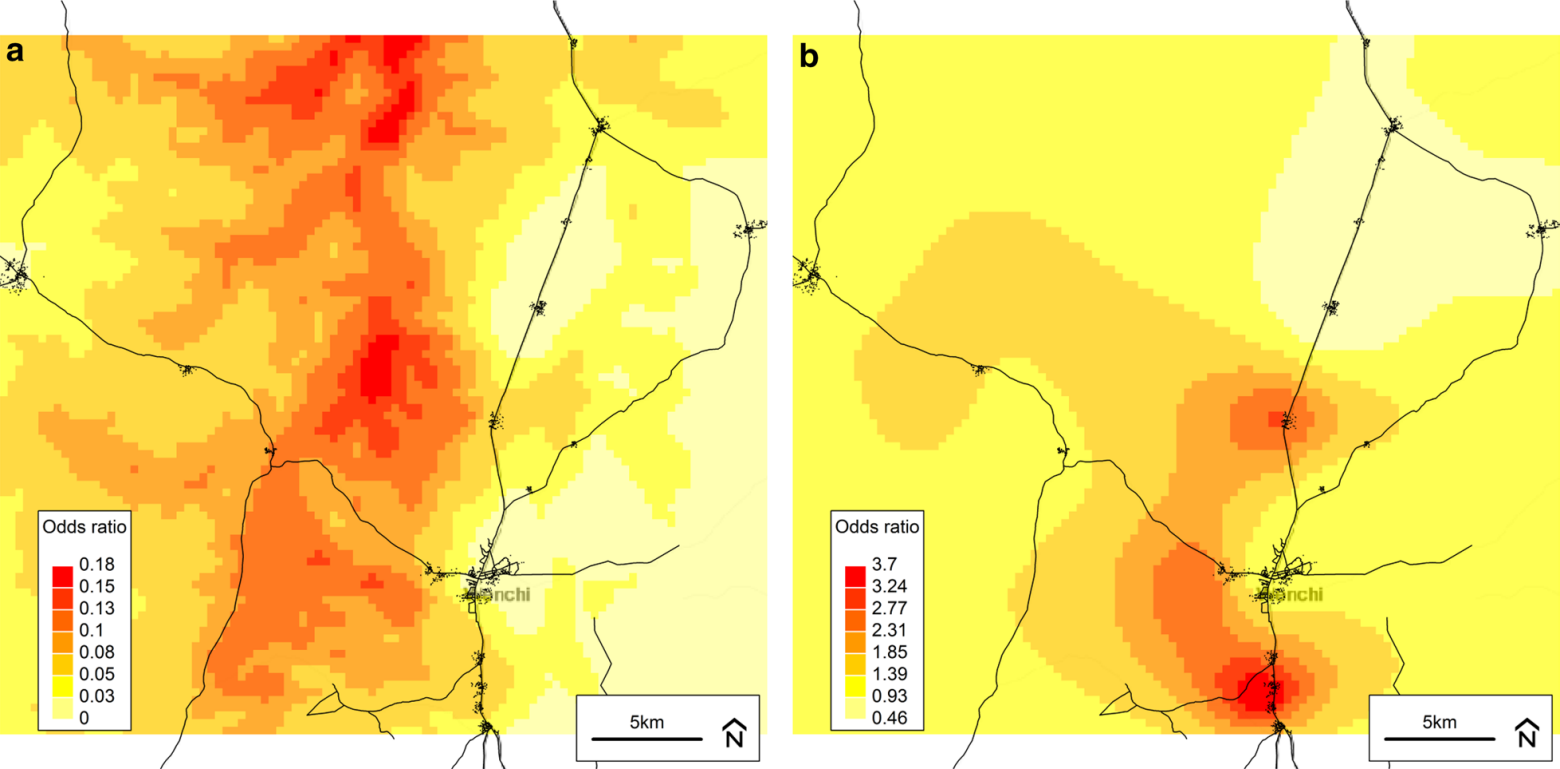
Plots from the final spatial model (Model 4) of parasitaemia with fever. **a** Predicted odds of malaria parasitaemia *with* concurrent fever (axillary temperature >37.5 °C—or history of reported fever within 48 h) at baseline from the final model (Model 4). **b** Residual spatial variation of malaria parasitaemia *with* concurrent fever at baseline from the final model (Model 4). *Darker colour* indicates higher risk. Background © Stamen Design

**Fig. 6:**
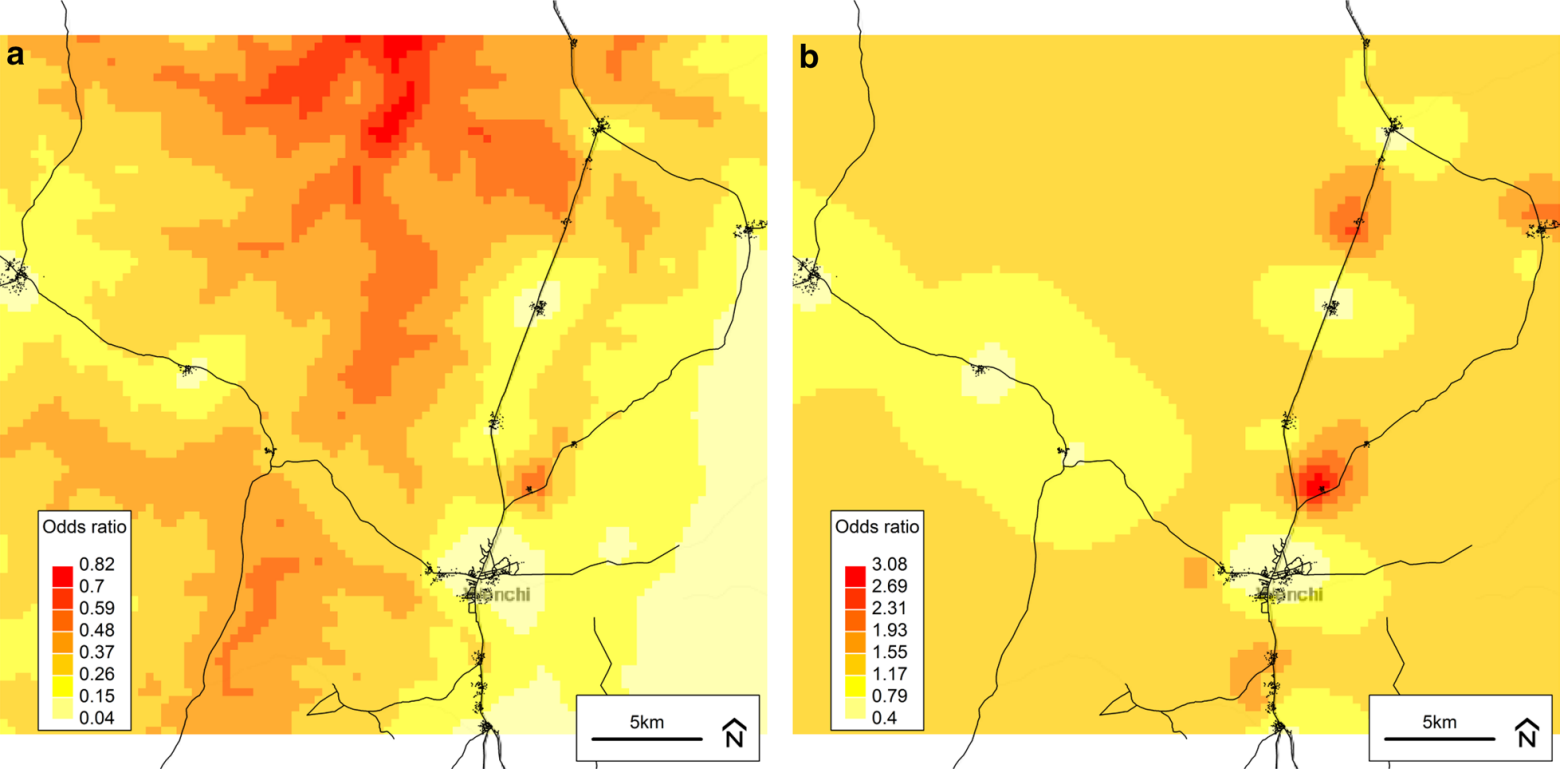
Plots from the final spatial model (Model 4) of all parasitaemia (with/without fever). **a** Predicted odds of all malaria parasitaemia (with or without fever) at baseline from the final model (Model 4). **b** Residual spatial variation of all malaria parasitaemia (with or without fever) at baseline from the final (Model 4). *Darker colour* indicates higher risk. Background © Stamen Design

**Fig. 7:**
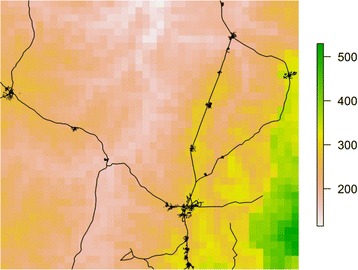
Plot of elevation changes (meters) across the study area. *Green colour* indicates lower elevation. *Black dots* represent trial compounds. *Lines* represent major roads

The predicted infection probabilities (odds) and residual spatial variation from all final models are illustrated in Figs. 3, 4, 5, 6. For infection status defined using parasitaemia (Figs. 3a, 5a, 6a), the relationship between infection risk and elevation was apparent particularly when compared to an elevation map of the study area (Fig. 7). For infection defined using CRP only, there appeared to be well-defined high- and low-risk areas that tended to cluster around villages (Fig. 4a). Unlike the infection definitions using parasitaemia, however, the plot of the spatial random effect for CRP only (Fig. 4b) was similar to that of the predicted odds (Fig. 4a), further supporting the observation that the covariates included in this final model did not explain a large amount of spatial variation in non-malaria infection.

Overall, the results from Model 5 confirmed those of Model 4, with the addition of maternal education being negatively associated with inflammation and/or parasitaemia (OR for ‘any’ education 0.79, 95 % CrI 0.63, 0.99), as well as parasitaemia with or without fever (OR 0.67, 95 % CrI 0.51, 0.87) (Table 3). All maximal models that included parasitaemia as part of the outcome definition also had a significant NDVI-LC interaction term, suggesting that outside of urbanized and built-up areas, each 0.1 ‘unit’ increase in greenness (with units ranging between 0.22 and 0.62) was associated with increases in the odds of infection of greater than 40 %.

**Table 3:** Results from the *maximal* spatial models (Model 5) of baseline infection status among 1943 Ghanaian children (2010)

Covariates	Odds ratios (95 % CrI)	Range parameter in km (95 % CrI)	Standard deviations of random effects (95 % CrI)	
			Spatial	Compound
(1) Inflammation and/or parasitaemia				
Intercept	0.252 (0.118, 0.543)	4.727 (1.971, 9.440)	0.420 (0.247, 0.733)	0.007 (0.004, 0.028)
Age per month (months)				
6–23	1.032 (1.010, 1.054)			
24–35	0.976 (0.939, 1.014)			
Sex (male reference)	1.116 (0.908, 1.372)			
Length-for-age z-score	0.915 (0.834, 1.003)			
Weight-for-length z-score	0.876 (0.785, 0.977)			
Asset score	1.032 (0.920, 1.156)			
Maternal education	0.791 (0.632, 0.991)			
Distance to health facility (km)	1.152 (1.053, 1.262)			
Elevation (m)	0.995 (0.990, 1.000)			
Urban/built up land (LC13)	0.965 (0.537, 1.718)			
Woody savannahs (LC8)	0.770 (0.258, 2.218)			
NDVI	0.628 (0.129, 2.972)			
NDVI*LC8	0.629 (0.249, 1.574)			
NDVI*LC13	5.237 (1.118, 26.00)			
Baseline iron status	1.128 (1.048, 1.217)			
(2) Inflammation without parasitaemia				
Intercept	0.141 (0.054, 0.353)	6.653 (2.576, 14.22)	0.489 (0.250, 0.961)	0.008 (0.004, 0.029)
Age per month (months)				
6–23	0.994 (0.967, 1.022)			
24–35	0.974 (0.921, 1.028)			
Sex (male reference)	1.168 (0.889, 1.535)			
Length-for-age z-score	1.007 (0.892, 1.137)			
Weight-for-length z-score	0.904 (0.783, 1.042)			
Asset score	0.994 (0.859, 1.149)			
Maternal education	1.126 (0.831, 1.539)			
Distance to health facility (km)	0.914 (0.810, 1.022)			
Elevation (m)	0.999 (0.993, 1.005)			
Urban/built up land (LC13)	1.035 (0.532, 1.988)			
Woody savannahs (LC8)	0.925 (0.152, 3.759)			
NDVI	1.172 (0.171, 7.074)			
NDVI*LC8	0.842 (0.270, 3.094)			
NDVI*LC13	1.038 (0.192, 6.405)			
Baseline iron status	1.043 (0.940, 1.145)			
(3) Parasitaemia with fever				
Intercept	0.006 (0.001, 0.023)	6.992 (2.160, 16.56)	0.675 (0.315, 1.387)	0.008 (0.004, 0.030)
Age per month (months)				
6–23	1.046 (1.006, 1.089)			
24–35	0.929 (0.859, 0.999)			
Sex (male reference)	1.276 (0.869, 1.877)			
Length-for-age z-score	0.911 (0.763, 1.084)			
Weight-for-length z-score	0.793 (0.639, 0.979)			
Asset score	1.042 (0.827, 1.312)			
Maternal education	0.917 (0.609, 1.392)			
Distance to health facility (km)	1.307 (1.114, 1.555)			
Elevation (m)	0.996 (0.987, 1.005)			
Urban/built up land (LC13)	1.227 (0.275, 4.872)			
Woody savannahs (LC8)	2.023 (0.300, 9.713)			
NDVI	0.128 (0.004, 3.152)			
NDVI*LC8	1.004 (0.235, 4.548)			
NDVI*LC13	32.80 (1.085, 1542)			
Baseline iron status	1.226 (1.104, 1.350)			
(4) All parasitaemia				
Intercept	0.093 (0.035, 0.241)	4.520 (1.494, 10.30)	0.519 (0.280, 0.953)	0.008 (0.004, 0.030)
Age per month (months)				
6–23	1.055 (1.028, 1.083)			
24–35	0.984 (0.941, 1.028)			
Sex (male reference)	1.026 (0.801, 1.313)			
Length-for-age z-score	0.874 (0.781, 0.977)			
Weight-for-length z-score	0.890 (0.779, 1.017)			
Asset score	1.065 (0.921, 1.230)			
Maternal education	0.665 (0.512, 0.866)			
Distance to health facility (km)	1.259 (1.130, 1.417)			
Elevation (m)	0.995 (0.988, 1.001)			
Urban/built up land (LC13)	0.729 (0.290, 1.733)			
Woody savannahs (LC8)	0.751 (0.217, 2.362)			
NDVI	0.375 (0.039, 3.146)			
NDVI*LC8	0.607 (0.223, 1.644)			
NDVI*LC13	11.19 (1.204, 127.0)			
Baseline iron status	1.129 (1.044, 1.222)			

Infection status definitions

1) Inflammation and/or parasitaemia (binary): 1 = CRP > 5 mg/L and/or any malaria parasitaemia, 0 = CRP ≤ 5 mg/L and absence of parasitaemia

2) Inflammation without parasitaemia (binary): 1 = CRP > 5 mg/L without malaria parasitaemia, 0 = CRP ≤ 5 mg/L without parasitaemia

3) Parasitaemia with fever (binary): 1 = any malaria parasitaemia with concurrent fever (axillary temperature > 37.5 °C) or history of reported fever (within 48 h), 0 = any malaria parasitaemia without concurrent fever or history of reported fever

4) All parasitaemia (binary): 1 = any malaria parasitaemia with/without fever, 0 = absence of parasitaemia with/without fever

Model prior shape = 1.117, model prior rate = 0.157

Baseline iron status = iron status at baseline, defined as serum ferritin concentration (µg/dL) corrected for CRP using the regression method and re-scaled by multiplying each corrected value by the inverse of the inter-quartile range

*CrI* credible interval, *NDVI* normalized difference vegetation index, averaged over the year 2010, centred by dividing by 1000 and subtracting 4, *NDVI8* interaction term between NDVI and LC = 8, *NDVI13* interaction term between NDVI and LC = 13

Most models demonstrated significant spatial random effects, indicating that there was residual variation in the odds of baseline infection across the study area, particularly when infection was defined as inflammation without parasitaemia. Comparatively, the compound random effects tended to be small with narrow 95 % CrI, indicating relatively low variability in infection risk between compounds. The range parameter from each model indicated that the distance at which the intervariable relationships started to decay (decreased covariance) ranged from 3.37 (95 % CrI 1.55, 6.82) to 7.55 (95 % CrI 3.08, 14.6) km in the final models (Model 4 set), and 4.52 (95 % CrI 1.49, 10.3) to 6.99 (95 % CrI 2.16, 16.6) km in the maximal models (Model 5 set).

## Discussion

The geostatistical analyses presented herein are the first to demonstrate spatial relationships for the risk of malaria and non-malaria infection, using standard and novel definitions, among children living in a malaria-endemic area with varying levels of iron status. In particular, elevation and distance to the nearest health facility were consistently associated with infection when it was defined using parasitaemia, either alone or in combination with CRP or fever. For example, in a final model, children with inflammation (CRP > 5 mg/L) and/or malaria parasitaemia at baseline were more likely to live farther from a health facility and at a lower elevation. Access to a healthcare system is generally considered to be a positive predictor of health, and this relationship is supported by other studies in malaria-endemic areas [16, 37, 38]. Assuming that malaria was the largest contributor to the prevalence of infection in this study’s population of Ghanaian children, the inverse relationship observed between elevation and infection status is consistent with other studies conducted in malaria-endemic areas showing a lower prevalence of malaria among populations living at higher elevations [39–42]. The prediction raster plots for the final models (Model 4 set) especially illustrate this relationship when compared to an elevation map of the study area (Fig. 7). The association between malaria and elevation is related to temperature, as the early stages of parasite development are sensitive to temperature and will be delayed or inhibited in colder environments, which are found at higher altitudes [35].

In terms of individual-level risk factors, children with parasitaemia (with or without high CRP) were more likely to be under 2 years of age, be stunted or wasted, and/or have higher ferritin concentration at baseline compared to children without infection. Infants tend to be at higher risk of infection due to immature immune systems [43], particularly at later stages of infancy when they begin to explore their environment, which increases the risk of exposure to pathogens. Both stunting (low length for age) and wasting (low weight for length) have also been associated with a higher risk of infection-related morbidity and mortality among children under 5 years of age in low- and middle-income countries [44, 45]. The only individual-level variable that remained significant across all definitions of infection in both Model sets 4 and 5 was baseline iron status. The relationship in all models was also positive, indicating that those with higher serum ferritin concentrations were more likely to have high CRP and/or parasitaemia. This is not surprising considering the well-known up-regulating effect of infection or inflammation on acute phase proteins like ferritin. Although baseline ferritin values were corrected for the effect of inflammation (using the regression method), the only biomarker available for this was CRP. During the early phase of the inflammatory response, CRP reaches its peak concentration within 24–48 h [11]. When the concentration of CRP declines, ferritin tends to remain elevated. Therefore, for more complete ferritin correction, an additional acute phase protein that corresponds to the late phase of the inflammatory response, such as alpha-1-acid glycoprotein (AGP), is needed [11]. In this case, it is possible that the prevalence of inflammation was underestimated, resulting in incomplete correction of ferritin and residual confounding.

Similar to Model 4, among the Model 5 set, infection defined using parasitaemia was the most informative in terms of identifying environmental and non-environmental relationships. Maternal education was negatively associated with infection status, which agrees with the generally reported finding that parental schooling has a positive influence on child health and nutrition status [46]. The significant NDVI-LC interaction term may be explained by the effect of land cover and land use on the survival and breeding behaviors of mosquitoes. Relationships between infection risk and vegetation type have been reported by others investigating the spatial risk factors of malaria in Ghana [47] and Indonesia [48]. For example, Krefis and colleagues found a lower incidence of malaria among children living in forested areas of rural Ghana (RR = 0.53), while those living in close proximity to cultivation had a higher risk of malaria [47]. Forest-type vegetation may be less likely to collect water where mosquitoes could breed and more easily infect those who live nearby [49]. On the other hand, areas that have been cleared or cultivated may be more likely to have standing water due to irrigation, certain topographical characteristics (e.g., slope), or poor drainage [39, 41, 50]. Appawu and colleagues demonstrated higher malaria transmission rates in irrigated communities compared to non-irrigated areas of the Kassena Nankana District of northern Ghana, where the land is primarily used for subsistence farming [50]. In the western highlands of Kenya, Cohen and colleagues found that households with confirmed malaria cases tended to be closer to areas with high wetness indices (predicted water accumulation), which were generated using hydrologic modelling of land surface water flow [39]. In the present analyses, the interaction between vegetation or greenness (NDVI) and ‘urbanized or built-up’ land cover type may reflect the differentiation between forested and cultivated land, and thus corresponding propensities for water to accumulate and create breeding grounds for *Anopheles* mosquitoes.

The significant spatial random effects observed in all models suggest that geographical distribution may be important to consider when assessing infection risk in a population. This was especially apparent when the final model outputs were plotted and compared to an elevation map, further demonstrating the utility of GIS and spatial analysis in exploring and communicating population health risks and characteristics. In some cases, particularly for infection defined using CRP only, mapping the spatial random effect from the final model suggested that the factors included in the analysis may not have fully explained the variation observed. While a larger sample size and/or geographical coverage may have allowed an additional insight into potential sources of spatial variation, this may also increase the risk of a type I error. An additional limitation pertains to CRP and its inability to capture the late phase of the inflammatory response (i.e., after 48 h), as this may have led to underestimation of non-malaria infection prevalence and thus an incomplete picture of spatial variability.

Similar to the spatial random effects, the model range remained relatively constant across models, although quite a bit smaller compared to other spatial analyses of malaria prevalence in sub-Saharan Africa. Ashton and colleagues used spatial modelling with a Bayesian framework to assess the spatial variation of malaria (*Plasmodiumfalciparum* and *Plasmodiumvivax*) among 5914 school children in Oromia Regional State, Ethiopia [51]. They described range as the distance at which similarities in climatic factors and ecology would be expected, and found that it was approximately 45 km in the *P. falciparum* model [51]. Although the outcome assessed by Ashton and colleagues was similar to that of the Ghana trial (*P. falciparum* parasitaemia), the range from each study may have been less comparable due to differences in key study characteristics. These included the measurement methods used (e.g., enzyme-linked immunosorbent assay versus microscopy in the Ghana trial for assessing malaria seroprevalence), the covariates included in the spatial models (e.g., environmental factors only versus a combination of environmental, individual- and household-level factors in the Ghana trial), and the size of the study area (284,500 km^2^ in Ethiopia versus 3200 km^2^ in Ghana).

Unlike the spatial random effects, the compound random effect observed in all models tended to be relatively small, suggesting that there was low additional variability in the outcome (infection status) across compounds. Since a compound may have consisted of more than one household, some spatial clustering of the outcome at the compound level was expected. Potential explanations for why this was not observed include: (1) the compounds within a village were in close proximity to each other, resulting in clustering at the village level rather than between compounds; and, (2) the small number of observations per compound reduced the opportunity for the outcome to cluster within compounds. Considering that the average cluster size was 1.3, the latter explanation was the more likely scenario.

A potential limitation of the present analyses was the use of straight-line (Euclidean) distance to estimate proximity to a health facility rather than an indicator of access by road, such as network distance. While, network distance may have more appropriately accounted for travel distance by vehicle or bicycle, it was not possible to calculate due to incomplete or missing vector information (e.g., misaligned junctions, missing or disconnected road segments). Another study conducted in the Brong-Ahafo region of Ghana, by Nesbitt and colleagues, compared different measures of travel impedance to estimate access to delivery care [52]. The authors encountered similar challenges with calculating network distance, and found that it was as informative as straight-line distance for determining geographical access in this area of rural Ghana [49]. In light of these findings, the use of Euclidean distance in the present analyses was considered to be justified. An additional limitation of the analyses presented here is the cross-sectional nature of the data, as it does not allow causality to be inferred or eliminate the risk of reverse causality. Further, the prevalence of protective behaviours (e.g. the use of insecticide treated nets or indoor residual spraying) was not assessed at baseline, and may have represented a source of unmeasured spatial variation.

## Conclusions

Determining the spatial dynamics of infection among children in a malaria-endemic area, without the use of invasive and costly measurement methods, may provide a means of locating high risk populations and identifying geographical areas where treatment and prevention strategies should be focused. Furthermore, considering the relationship between inflammation and iron homeostasis, the maps of infection risk presented here could also inform the geographical distribution of iron deficiency risk, or at least help to identify areas where extra caution should be used when providing iron interventions to infants and young children. Future research should include longitudinal analyses to examine the co-variation in geo-spatial factors associated with infection status over time, and to further explore the potential importance of baseline effects.

## Additional file

10.1186/s12936-016-1388-1 Results from spatial Models 1–3 of baseline infection status. Three tables summarizing the set of results from Model 1 (individual-level variables), Model 2 (household-level variables), and Model 3 (satellite-derived variables) for all four infection definitions.

## Abbreviations

AGP: alpha-1-acid glycoprotein
CBC: complete blood count
CRP: C-reactive protein
GHS: Ghana Health Service
GLGM: generalized linear geostatistical model
GPS: global positioning system
IGBP: International Geosphere Biosphere Programme
INLA: integrated nested Laplace approximation
KHRC: Kintampo Health Research Centre
LC: land cover type
LMIC: low- and middle-income countries
LPDAAC: Land Processes Distributed Active Archive Center
NDVI: naturalized difference vegetation index
UTM: Universal Transverse Mercator
USGS: US Geological Survey
WHO: World Health Organization
