# Supplementary material for: Geo-spatial factors associated with infection risk among young children in rural Ghana: a secondary spatial analysis
Source: Malar J. 2016 Jul 8;15:349. doi: 10.1186/s12936-016-1388-1 (PMC4938940; doi:10.1186/s12936-016-1388-1)
Supplement: Supplementary file 1 — 10.1186/s12936-016-1388-1 Results from spatial Models 1–3 of baseline infection status. Three tables summarizing the set of results from Model 1 (individual-level variables), Model 2 (household-level variables), and Model 3 (satellite-derived variables) for all four infection definitions. [file 12936_2016_1388_MOESM1_ESM.pdf]

Table S1: Spatial models of individual-level factors associated with baseline infection status[1] among 1943 Ghanaian children (Brong-Ahafo Region, March-April 2010)

Covariates	Odds ratios (95% CrI)		Range parameter in km (95% CrI)	Standard deviations of random effects (95% CrI)	
				Spatial	Compound
**1) Inflammation and/or parasitemia**					
Intercept	0.703	(0.459, 1.099)	3.865	0.639	0.008
Age per month			(1.934,	(0.434,	(0.031,
6-23 months	1.031	(1.010, 1.051)	6.931)	0.961)	0.004)
24-35 months	0.968	(0.933, 1.003)			
Sex (male reference)	1.079	(0.888, 1.310)			
Length-for-age z-score	0.904	(0.829, 0.985)			
Weight-for-length z-score	0.890	(0.803, 0.986)			
Baseline iron status	1.142	(1.065, 1.229)			
**2) Inflammation without parasitemia**					
Intercept	0.125	(0.079, 0.196)	7.772	0.456	0.008
Age per month			(3.074,	(0.248,	(0.030,
6-23 months	0.994	(0.968, 1.020)	16.56)	0.846)	0.004)
24-35 months	0.965	(0.914, 1.016)			
Sex (male reference)	1.184	(0.912, 1.537)			
Length-for-age z-score	1.006	(0.896, 1.128)			
Weight-for-length z-score	0.892	(0.778, 1.021)			
Baseline iron status	1.023	(0.924, 1.120)			
**3) Parasitemia with fever**					
Intercept	0.064	(0.028, 0.131)	5.772	0.998	0.008
Age per month			(1.823,	(0.650,	(0.030,
6-23 months	1.037	(1.001, 1.076)	16.81)	1.517)	0.004)
24-35 months	0.936	(0.871, 1.001)			
Sex (male reference)	1.138	(0.800, 1.619)			

Length-for-age z-score	0.960	(0.818, 1.123)			
Weight-for-length z-score	0.820	(0.676, 0.992)			
Baseline iron status	1.226	(1.114, 1.343)			
**4) All parasitemia**					
Intercept	0.443	(0.244, 0.834)	3.295	1.036	0.008
Age per month			(2.002,	(0.725,	(0.033,
6-23 months	1.053	(1.028, 1.079)	5.268)	1.500)	0.004)
24-35 months	0.980	(0.940, 1.021)			
Sex (male reference)	0.973	(0.771, 1.228)			
Length-for-age z-score	0.868	(0.781, 0.962)			
Weight-for-length z-score	0.914	(0.807, 1.035)			
Baseline iron status	1.151	(1.067, 1.242)			

[1]Infection status definitions:

1) Inflammation and/or parasitemia (binary): 1 = CRP > 5 mg/L and/or any malaria parasitemia, 0 = CRP ≤ 5 mg/L and absence of parasitemia;

2) Inflammation without parasitemia (binary): 1 = CRP > 5 mg/L without malaria parasitemia, 0 = CRP ≤ 5 mg/L without parasitemia;

3) Parasitemia with fever (binary): 1 = any malaria parasitemia with concurrent fever (axillary temperature >37.5^0C) or history of reported fever (within 48 hours), 0 = any malaria parasitemia without concurrent fever or history of reported fever;

4) All parasitemia (binary): 1 = any malaria parasitemia with/without fever, 0 = absence of parasitemia with/without fever

Model prior shape = 1.117, model prior rate = 0.157

CrI = credible interval

Baseline iron status = iron status at baseline, defined as serum ferritin concentration corrected for CRP using the regression method (Namaste et al.)

Table S2: Spatial models of household-level factors associated with infection status[1] among 1943 Ghanaian children (Brong-Ahafo Region, March-April 2010)

Covariates	Odds ratios (95% CrI)		Range parameter in km (95% CrI)	Standard deviations of random effects (95% CrI)	
				Spatial	Compound
**1) Inflammation and/or parasitemia**					
Intercept	0.541	(0.322, 0.866)	4.162	0.538	0.007
Asset score	1.050	(0.938, 1.174)	(1.828,	(0.346,	(0.028,
Maternal education	0.752	(0.603, 0.939)	8.227)	0.866)	0.004)
Distance to health facility (km)	1.152	(1.055, 1.277)			
**2) Inflammation without parasitemia**					
Intercept	0.183	(0.115, 0.285)	7.277	0.416	0.008
Asset score	0.991	(0.860, 1.141)	(2.661,	(0.221,	(0.029,
Maternal education	1.107	(0.821, 1.504)	16.26)	0.795)	0.004)
Distance to health facility (km)	0.918	(0.839, 1.001)			
**3) Parasitemia with fever**					
Intercept	0.033	(0.013, 0.071)	8.068	0.867	0.008
Asset score	1.079	(0.868, 1.337)	(3.180,	(0.460,	(0.029,
Maternal education	0.893	(0.602, 1.337)	16.33)	1.640)	0.004)
Distance to health facility (km)	1.241	(1.076, 1.458)			
**4) All parasitemia**					
Intercept	0.228	(0.115, 0.420)	3.795	0.721	0.008
Asset score	1.085	(0.942, 1.249)	(1.604,	(0.479,	(0.031,
Maternal education	0.638	(0.494, 0.827)	7.813)	1.101)	0.004)
Distance to health facility (km)	1.248	(1.113, 1.425)			

[1]Infection status definitions:

1) Inflammation and/or parasitemia (binary): 1 = CRP > 5 mg/L and/or any malaria parasitemia, 0 = CRP ≤ 5 mg/L and absence of parasitemia;

2) Inflammation without parasitemia (binary): 1 = CRP > 5 mg/L without malaria parasitemia, 0 = CRP ≤ 5 mg/L without parasitemia;

3) Parasitemia with fever (binary): 1 = any malaria parasitemia with concurrent fever (axillary temperature >37.5^{0}C) or history of reported fever (within 48 hours), 0 = any malaria parasitemia without concurrent fever or history of reported fever;

4) All parasitemia (binary): 1 = any malaria parasitemia with/without fever, 0 = absence of parasitemia with/without fever

Model prior shape = 1.117, model prior rate = 0.157

CrI = credible interval

Table S3: Spatial models of satellite-derived geo-spatial factors associated with baseline infection status[1] among 1943 Ghanaian children (Brong-Ahafo Region, March-April 2010)

Covariates	Odds ratios (95% CrI)		Range parameter in km (95% CrI)	Standard deviations of random effects (95% CrI)	
				Spatial	Compound
**1) Inflammation and/or parasitemia**					
Intercept	0.561	(0.298, 1.017)	5.827	0.637	0.008
Elevation (m)	0.991	(0.985, 0.997)	(2.844,	(0.398,	(0.030,
Urban/built up land (LC13)	0.810	(0.466, 1.387)	10.42)	1.043)	0.004)
Woody savannahs (LC8)	0.744	(0.261, 2.081)			
NDVI	0.722	(0.151, 3.318)			
NDVI*LC8	0.683	(0.263, 1.784)			
NDVI*LC13	3.095	(0.696, 14.88)			
**2) Inflammation without parasitemia**					
Intercept	0.117	(0.063, 0.215)	6.663	0.486	0.008
Elevation (m)	1.001	(0.995, 1.006)	(2.706,	(0.260,	(0.029,
Urban/built up land (LC13)	1.135	(0.614, 2.067)	13.84)	0.915)	0.004)
Woody savannahs (LC8)	0.865	(0.145, 3.424)			
NDVI	1.019	(0.183, 5.401)			
NDVI*LC8	0.805	(0.272, 2.637)			
NDVI*LC13	1.436	(0.299, 7.321)			
**3) Parasitemia with fever**					
Intercept	0.043	(0.013, 0.112)	9.251	1.062	0.008
Elevation (m)	0.990	(0.981, 0.999)	(3.233,	(0.584,	(0.030,
Urban/built up land (LC13)	0.757	(0.213, 2.399)	19.85)	1.985)	0.004)
Woody savannahs (LC8)	1.268	(0.192, 5.738)			
NDVI	0.485	(0.020, 8.294)			
NDVI*LC8	0.729	(0.179, 3.308)			
NDVI*LC13	4.835	(0.255, 155.3)			

4) All parasitemia

Intercept	0.360	(0.143, 0.856)	4.396	0.982	0.008
Elevation (m)	0.990	(0.981, 0.999)	(2.034,	(0.643,	(0.032,
Urban/built up land (LC13)	0.488	(0.184, 1.192)	8.847)	1.498)	0.004)
Woody savannahs (LC8)	0.688	(0.204, 2.197)			
NDVI	0.325	(0.022, 4.002)			
NDVI*LC8	0.828	(0.261, 2.711)			
NDVI*LC13	4.884	(0.349, 88.71)			

[1]Infection status definitions:

1) Inflammation and/or parasitemia (binary): 1 = CRP > 5 mg/L and/or any malaria parasitemia, 0 = CRP ≤ 5 mg/L and absence of parasitemia;

2) Inflammation without parasitemia (binary): 1 = CRP > 5 mg/L without malaria parasitemia, 0 = CRP ≤ 5 mg/L without parasitemia;

3) Parasitemia with fever (binary): 1 = any malaria parasitemia with concurrent fever (axillary temperature >37.5^{0}C) or history of reported fever (within 48 hours), 0 = any malaria parasitemia without concurrent fever or history of reported fever;

4) All parasitemia (binary): 1 = any malaria parasitemia with/without fever, 0 = absence of parasitemia with/without fever

Model prior shape = 1.117, model prior rate = 0.157

CrI = credible interval

NDVI = normalized difference vegetation index, averaged over 2010 yearly values, centered by dividing by 1000 and subtracting 4
